# Dentinal Microcrack Formation after Root Canal Instrumentation by XP-Endo Shaper and ProTaper Universal: A Microcomputed Tomography Evaluation

**DOI:** 10.1155/2020/4030194

**Published:** 2020-04-08

**Authors:** Sarah M. Alkahtany, Ebtissam M. Al-Madi

**Affiliations:** Division of Endodontics, Department of Restorative Dentistry, College of Dentistry, King Saud University, P.O. Box 5967, Riyadh 11432, Saudi Arabia

## Abstract

**Aim:**

To evaluate dentinal microcrack formation on root canals instrumented, continuously in the body temperature, with XP-endo shaper (XPES) and ProTaper Universal (PTU), by means of microcomputed tomographic (micro-CT) analysis. *Methodology*. Nineteen mesial roots with two separate canals (Vertucci Type IV) of extracted mandibular molars were used in this study. The root canals (*N* = 38) were divided into 2 groups. Group 1 (*n* = 19): all MB canals were instrumented with XPES. Group 2 (*n* = 19): all ML canals were instrumented with PTU. All roots were scanned with micro-CT before and after instrumentation. Two precalibrated examiners evaluated the cross-sectional images of each sample with DataViewer program. The dentinal microcracks (complete and incomplete) were counted in each third of the root for the preinstrumentation and the postinstrumentation images. Wilcoxin signed-rank and Mann–Whitney *U* tests were used for statistical analysis at a significance level of *P* < 0.05.

**Results:**

The number of microcracks increased significantly (*P* < 0.05) after instrumentation with XPES in the middle and cervical thirds. The number of microcracks increased significantly (*P* < 0.05) after instrumentation with PTU in the cervical third only. There was no significant difference between the groups in the cervical and apical thirds. In the middle third, the XPES induced more incomplete microcracks than PTU (*P* < 0.05).

**Conclusion:**

Within the limitations of this study, there was no significant difference in the dentinal microcrack formation between XPES and PTU in the apical and cervical thirds of the root. However, XPES instrumentation induced more incomplete microcracks than PTU in the middle third of human roots.

## 1. Introduction

Disinfection of the root canal system is essential for successful root canal treatment [1]. The antibacterial effect of sodium hypochlorite cannot reach all bacteria in the dentinal tubules [2]. Therefore, root canal mechanical enlargement is required to ensure the removal of infected dentine [3]. Cleaning and shaping procedures have significantly improved with the use of NiTi rotary instruments [4]. However, NiTi files might lead to dentinal defects and can induce microcracks in the dentinal walls of the root canal [5].

Microcomputed tomography (micro-CT) is the method of choice to evaluate and assess dentinal defects and microcracks induced by root canal instrumentation with different systems. It allows the investigators to evaluate hundreds of axial sections per tooth to accurately detect the exact location of a microcrack [6]. Furthermore, micro-CT is a nondestructive and noninvasive technique to obtain two-dimensional and three-dimensional images of any tooth [7]. It enables scanning of the same sample for multiple tests without damage allowing each sample to be used as its own control [8].

The ProTaper Universal (PTU) (Dentsply Tulsa Dental Specialties, Tulsa, OK) Ni–Ti rotary system is one of the most commonly used files. It is machined from conventional superelastic (SE) austenitic Ni-Ti wire. It features a variable taper over the entire cutting blade length with convex triangular cross sections. This file design can help clinicians properly instrument and flare canals with anatomical difficulties [9]. However, PTU has been reported to be associated with a high incidence of microcrack formation [5, 10–15].

The XP-endo shaper (XPES) (FKG Dentaire, Switzerland) is made of MaxWire alloy, a martensite-austenite-electropolish thermomechanically treated NiTi alloy. This file will curve on exposure to body temperature, due to the phase transformation from the M-phase (martensitic state) to the A-phase (austenitic state) [16]. The manufacturer claims that the flexibility and preset shape enable the XPES to contract and expand within the canal itself and to reach areas that conventional files cannot access. Furthermore, XPES has an ISO size 30 diameter and 0.01 taper, which could minimize the physical stresses on the canal dentinal wall. Recent publications reported that the XPES system performed well in root canal instrumentation including severely curved canals but leaves untouched dentinal wall areas [17, 18].

Previous studies reported that XPES instrumentation will cause no or few dentinal microcracks compared with other NiTi rotary systems [19–21]. None of these studies exposed the XPES files to body temperature during the instrumentation. Therefore, in the present study, we aim to evaluate dentinal microcrack formation on root canals instrumented, continuously in the body temperature, with the XP-endo shaper (XPES) and ProTaper Universal (PTU), by means of microcomputed tomographic (micro-CT) analysis.

## 2. Materials and Methods

This research was conducted in King Saud University, Riyadh, Saudi Arabia. The research protocol was approved by the Institutional Review Board (E-17-2646).

### 2.1. Specimen Selection

Thirty-six extracted mandibular molars were collected, sterilized in 10% buffered formalin. All teeth were decoronated, and the lengths of roots were standardized to 16 mm. The roots were split at the furcation area by using ISOMET 2000 PRECISION SAW (Buehler, USA). Straight and angulated conventional radiographs were taken for all mesial roots to verify the inclusion criteria. The inclusion criteria were as follows: mesial roots with two separate canals (Vertucci Type IV), free from calcifications and pulp stones, and a root curvature between 10° and 30° as verified by measurement of Schneider [22]. Finally, nineteen mesial roots with thirty-eight root canals were selected and included in this study (*N* = 38). Each root was mounted in clear acrylic block with a mark on the buccal side. This mark will assist the positioning of each sample in the same orientation for pre- and postinstrumentation micro-CT scan.

### 2.2. Preinstrumentation Micro-CT Scan

The roots were scanned before instrumentation (preinstrumentation scan) with Skyscan1172 (Bruker, USA) 100 kV/98 *μ*A with a Hamamatsu 10-MP camera. The camera pixel size was 11.40 *μ*m with median filtering and flat-field correction.

### 2.3. Sample Preparation

The working length of all the canals was determined and confirmed by radiographs. Coronal flaring was performed for all canals by using a Gates-Glidden size 2, followed by preparation of a glide path for all canals with hand files (K file) up to size 15. RC-Prep® (Premier Dental, USA) was used as the lubricant. Canals were irrigated with 1 mL of 5% sodium hypochlorite (NaOCl) before and after each file. The mesial root canals (*N* = 38) were divided into two groups:

  XPES group (*n* = 19): mesiobuccal canals were instrumented with the XPES system. The files were mounted on the X-smart handpiece (Dentsply Tulsa Dental Specialties, Tulsa, OK) and used at a speed of 800 rpm and 1 N·cm, according to the manufacturer's instructions. Each file was used in 3 gentle strokes to the full working length. RC-Prep® was used as the lubricant, and 1 mL of 5% NaOCl was used for irrigation after instrumentation.
  PTU group (*n* = 19): mesiolingual canals were instrumented with the PTU system. The files were mounted on the X-smart handpiece and used at a speed of 300 rpm and 1 N·cm, according to the manufacturer's instructions. The canals were instrumented with S1, S2, F1, F2, and F3. Each file was lubricated with RC-Prep®, and 1 mL of 5% NaOCl was used for irrigation after each file.

All the roots were submerged in a water bath at 37°C during instrumentation to mimic the body temperature. All files were used 3 times and then discarded to prevent separation.

### 2.4. Postinstrumentation Micro-CT Scan

After instrumentation, all samples were scanned again with Skyscan1172 (100 kV/98 *μ*A) with the Hamamatsu 10-MP camera. The camera pixel size was 11.40 *μ*m with median filtering and flat-field correction.

### 2.5. Dentinal Microcrack Evaluation

Two precalibrated examiners evaluated cross-sectional images of each sample with the DataViewer program (version 1.5.2.4, Bruker, USA). Each reconstructed root image was divided into thirds (cervical, middle, and apical). Next, dentinal microcracks (complete and incomplete) were counted in each third of the root in the preinstrumentation and postinstrumentation images.

### 2.6. Statistical Analysis

Inter-rater reliability was assessed by calculating the percentage agreement between the two examiners. The data were analyzed statistically and summarized using the chi-squared test to calculate the percentage of microcracks in each group. A comparison between the number of microcracks before and after instrumentation was performed with the Wilcoxon signed-rank test. The Mann–Whitney *U* test was used to compare the differences between the XPES and PTU groups at a significance level of *P* < 0.05 by using IBM SPSS® Statistics.

## 3. Results

**Figure 1:**
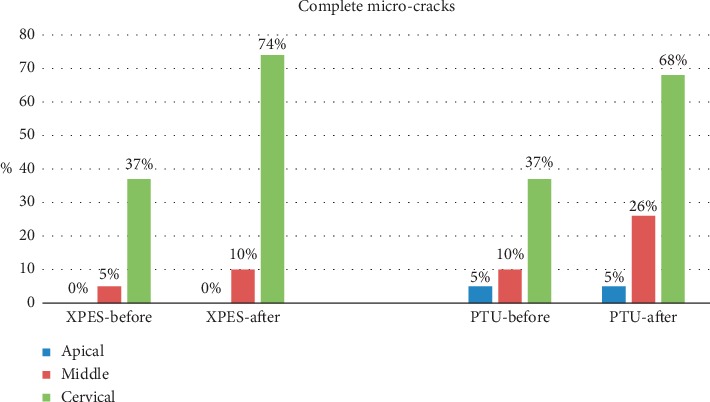
Percentage of complete microcracks before and after instrumentation in the XPES and PTU groups.

**Figure 2:**
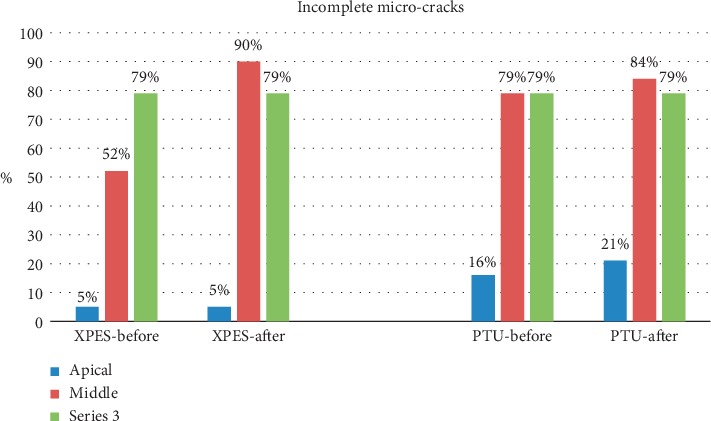
Percentage of incomplete microcracks before and after instrumentation in the XPES and PTU groups.

A total of 17,430 cross sections were evaluated by two examiners. The inter-rater percentage agreement was excellent (90%). The percentages of complete microcracks in each group are illustrated in Figure 1, while the percentages of incomplete microcracks in each group are illustrated in Figure 2. The total number of complete and incomplete microcracks in each group before and after instrumentation is shown in Table 1.

**Figure 3:**
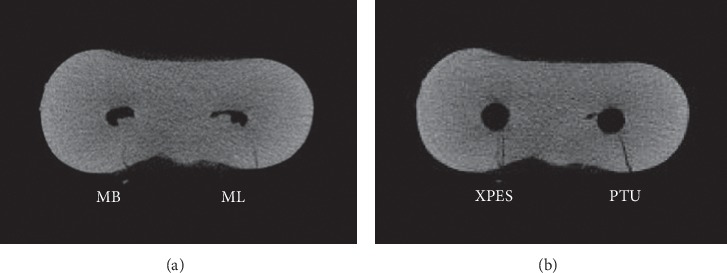
Micro-CT cross-sectional image of the cervical third of mandibular molar mesial root: (a) Preinstrumentation; (b) postinstrumentation. The MB canal (left) instrumented with XPES and the ML canal (right) instrumented with PTU. The microcracks that appeared in the postinstrumentation image for both canals were the propagation of previous microcracks observed in the preinstrumentation image.

**Figure 4:**
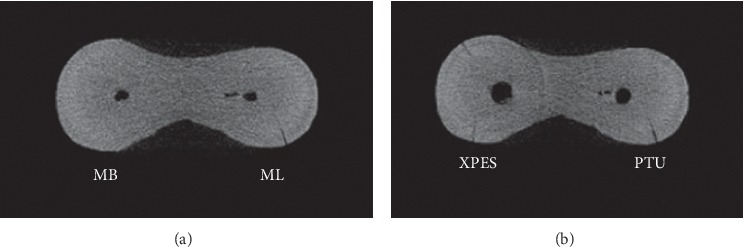
Micro-CT cross-sectional image of the middle third of mandibular molar mesial root: (a) preinstrumentation; (b) postinstrumentation. The MB canal instrumented with XPES (left) and the ML canal instrumented with PTU (right). One microcrack that appeared in the postinstrumentation image in the ML canal (PTU group) was the same as the preexisting microcrack observed in the preinstrumentation image. However, in the XPES group, two new incomplete microcracks were observed in the postinstrumentation image.

**Figure 5:**
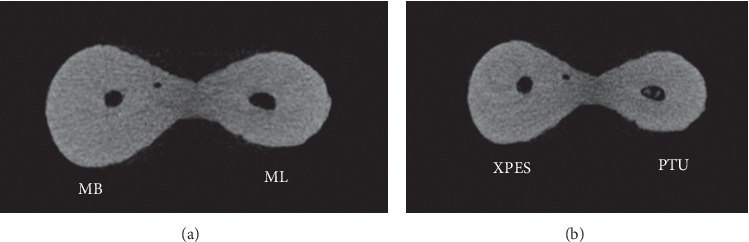
Micro-CT cross-sectional image of the apical third of mandibular molar mesial root. (a) Preinstrumentation; (b) postinstrumentation. The MB canal instrumented with XPES (left) and ML canal instrumented with PTU (right). No microcracks could be detected in the pre- or postinstrumentation images.

Most micro-CT images showed microcracks in the cervical and middle thirds before the instrumentation (52%–79% incomplete and 5%–37% complete microcracks). In general, the number of microcracks in the cervical and middle thirds increased after instrumentation (Figures 3 and 4). The number of complete microcracks in the cervical third increased significantly (*P* < 0.05) after instrumentation in both XPES and PTU groups, while the number of incomplete microcracks in the middle third increased significantly (*P* < 0.05) after instrumentation only in the XPES group (Figure 4). The apical third in all groups did not show any complete microcracks and only a few incomplete microcracks (Figure 5). There was no significant increase in the number of microcracks in the apical third after instrumentation.

There were no significant intergroup differences in the number of incomplete microcracks in the cervical and apical thirds. However, in the middle third, the XPES induced significantly more incomplete microcracks than PTU.

## 4. Discussion

Endodontic practice aims to restore and preserve remaining natural dentition using safe instruments and techniques. However, mechanical root canal preparation might induce microcracks that could propagate to root fractures, leading to poor prognosis [11, 23]. Therefore, it is essential to assess the safety of any new file including the incidence of dentinal microcrack formation.

The present study used micro-CT evaluation to compare the dentinal microcrack formation on root canals instrumented with PTU and XPES. The results showed that most of the microcracks seen in postinstrumentation images were present in the preinstrumentation images. However, many postinstrumentation complete microcracks were incomplete microcracks in the preinstrumentation images. According to Stringhet et al., this change in the type of microcracks was due to root canal lumen enlargement rather than a true propagation of the previous incomplete microcrack [6]. No attempt was made in this study to measure the length of the microcracks. Only the number and the type of microcracks (complete or incomplete) were evaluated. Our results demonstrated that instrumentation with both tested files induced a few new microcracks in root canal walls. However, the increase in the number of microcracks was statistically significant only in the middle and cervical thirds.

There was a significant increase in the number of complete microcracks after instrumentation in the cervical third in both groups. This could be due to the coronal flaring with Gates–Glidden drills or due to rotary file movement. Furthermore, the results showed a significant increase in the number of incomplete microcracks in the middle third in the XPES group in comparison with the PTU group. This might be attributed to the high-speed rotation (800 rpm) of the file and/or the nature of the movement of XPES. When the file is exposed to body temperature, it can contract and expand while rotating inside the canal due to its flexibility and preset shape. During our experiment, the operator experienced more vibration while using XPES compared with PTU.

Our results disagree with the findings of the previous studies. Bayram et al. compared the number of microcracks induced by XPES and ProTaper Gold (PTG), and their results showed that the PTG system significantly increased the incidence of microcracks, while the XPES system did not induce any new dentinal microcracks [19]. Ugur Aydin et al. compared the percentages of new microcracks formed after instrumentation with Reciproc Blue, XPES, and WaveOne Gold. Their investigation concluded that none of the used rotary systems caused new microcracks formation or propagation of existing microcracks [20]. Furthermore, our results contradict the findings of Aksoy et al.; their study concluded that PTU induced more microcracks than XPES [21]. This disagreement might be attributed to differences in methodology; in our study, the file was tested and used at body temperature (37°C) throughout the instrumentation. However, in the previous studies, the file was exposed to body temperature only once before the instrumentation.

In the present investigation, we did not use the fresh cadaveric model suggested by De-Deus et al. because this model was not readily available in most institutes, inluding ours [24]. Therefore, human extracted teeth were used, although a previous investigation reported that extracted teeth showed some microcracks induced by the extraction procedure itself [25]. In our study, all preexisted microcracks were recorded in the preinstrumentation micro-CT images, and they were not related to the root canal preparation.

The results of this study should be interpreted with caution due to some limitations. All extracted teeth were decoronated before the instrumentation; this was performed to limit the variation between the root lengths. Furthermore, the sectioned roots were mounted directly in hard acrylic blocks. However, these conditions do not represent a real clinical scenario. Moreover, the root canals were not randomly distributed between the groups; this might cause sampling bias. Finally, our results might be affected by the use of Gates-Glidden for preflaring of root canals. The solo use of an endodontic file inside the root canal is recommended. We recommend future investigators to use soft material around the teeth before mounting them in acrylic blocks and to complete the root canal instrumentations through the crowns without any sectioning to have more realistic results.

## 5. Conclusion

Within the limitations of this study, there was no significant difference in the dentinal microcrack formation between XP-endo shaper (XPES) and ProTaper Universal (PTU) in the apical and cervical thirds of the root. However, XPES instrumentation induced more incomplete microcracks than PTU in the middle third of human roots.

## Figures and Tables

**Table 1:** The number of complete and incomplete microcracks before and after instrumentation with XPES and PTU.

Type of microcrack	Groups		Number of microcracks		
			Apical	Middle	Cervical
Complete	XPES	Before	0	1	8
		After	0	3	19
		*P* value	1.000	0.157	0.0001∗
	PTU	Before	1	2	11
		After	1	5	20
		*P* value	1.000	0.083	0.011∗
	XPES vs. PTU	*P* value	1.000	0.636	0.685

Incomplete	XPES	Before	3	23	41
		After	3	41	45
		*P* value	1.000	0.0001∗	0.465
	PTU	Before	8	27	35
		After	10	37	37
		*P* value	0.317	0.317	0.627
	XPES vs. PTU	*P* value	0.317	0.025∗	0.662

∗ Statistically significant difference.

## Data Availability

The micro-CT images used to support the findings of this study are available from the corresponding author upon request.

## References

[1] Neves M. A. S., Provenzano J. C., Rôças I. N., Siqueira J. F. (2016). Clinical antibacterial effectiveness of root canal preparation with reciprocating single-instrument or continuously rotating multi-instrument systems. *Journal of Endodontics*.

[2] Haapasalo M., Ørstavik D. (1987). In vitro infection and of dentinal tubules. *Journal of Dental Research*.

[3] Wong D. T. S., Cheung G. S. P. (2014). Extension of bactericidal effect of sodium hypochlorite into dentinal tubules. *Journal of Endodontics*.

[4] Kuzekanani M. (2018). Nickel-titanium rotary instruments: development of the single-file systems. *Journal of International Society of Preventive and Community Dentistry*.

[5] Capar I. D., Arslan H., Akcay M., Uysal B. (2014). Effects of ProTaper universal, ProTaper next, and HyFlex instruments on crack formation in dentin. *Journal of Endodontics*.

[6] Stringheta C. P., Pelegrine R. A., Kato A. S. (2017). Micro-computed tomography versus the cross-sectioning method to evaluate dentin defects induced by different mechanized instrumentation techniques. *Journal of Endodontics*.

[7] Nielsen R. B., Alyassin A. M., Peters D. D., Carnes D. L., Lancaster J. (1995). Microcomputed tomography: an advanced system for detailed endodontic research. *Journal of Endodontics*.

[8] Swain M. V., Xue J. (2009). State of the art of micro-CT applications in dental research. *International Journal of Oral Science*.

[9] Ruddle C. J. (2001). The ProTaper endodontic system: geometries, features, and guidelines for use. *Dentistry Today*.

[10] Karatas E., Gunduz H. A., Kirici D. O., Arslan H. (2016). Incidence of dentinal cracks after root canal preparation with ProTaper gold, profile vortex, F360, Reciproc and ProTaper universal instruments. *International Endodontic Journal*.

[11] Abou El Nasr H. M., Abd El Kader K. G. (2014). Dentinal damage and fracture resistance of oval roots prepared with single-file systems using different kinematics. *Journal of Endodontics*.

[12] Ashraf F., Shankarappa P., Misra A., Sawhney A., Sridevi N., Singh A. (2016). A stereomicroscopic evaluation of dentinal cracks at different instrumentation lengths by using different rotary files (ProTaper universal, ProTaper next, and HyFlex CM): an ex vivo study. *Scientifica*.

[13] Shori D. D., Shenoi P. R., Baig A. R., Kubde R., Makade C., Pandey S. (2015). Stereomicroscopic evaluation of dentinal defects induced by new rotary system: “ProTaper NEXT. *Journal of Conservative Dentistry*.

[14] Zhou X., Jiang S., Wang X., Wang S., Zhu X., Zhang C. (2015). Comparison of dentinal and apical crack formation caused by four different nickel-titanium rotary and reciprocating systems in large and small canals. *Dental Materials Journal*.

[15] Nishad S. V., Shivamurthy G. B. (2018). Comparative analysis of apical root crack propagation after root canal preparation at different instrumentation lengths using ProTaper universal, ProTaper next and ProTaper gold rotary files: an in vitro study. *Contemporary Clinical Dentistry*.

[16] Zupanc J., Vahdat-Pajouh N., Schäfer E. (2018). New thermomechanically treated NiTi alloys - a review. *International Endodontic Journal*.

[17] Alfadley A., Alrajhi A., Alissa H. (2020). Shaping ability of XP endo shaper file in curved root canal models. *International Journal of Dentistry*.

[18] Velozo C., Albuquerque D. (2019). Microcomputed tomography studies of the effectiveness of XP-endo shaper in root canal preparation: a review of the literature. *Scientific World Journal*.

[19] Bayram H. M., Bayram E., Ocak M., Uygun A. D., Celik H. H. (2017). Effect of ProTaper gold, self-adjusting file, and XP-endo shaper instruments on dentinal microcrack formation: a micro-computed tomographic study. *Journal of Endodontics*.

[20] Ugur Aydin Z., Keskin N. B., Ozyurek T. (2019). Effect of Reciproc blue, XP-endo shaper, and WaveOne gold instruments on dentinal microcrack formation: a micro-computed tomographic evaluation. *Microscopy Research and Technique*.

[21] Aksoy Ç., Keriş E. Y., Yaman S. D., Ocak M., Geneci F., Çelik H. H. (2019). Evaluation of XP-endo shaper, Reciproc blue, and ProTaper universal NiTi systems on dentinal microcrack formation using micro-computed tomography. *Journal of Endodontics*.

[22] Schneider S. W. (1971). A comparison of canal preparations in straight and curved root canals. *Oral Surgery, Oral Medicine, Oral Pathology*.

[23] Bier C. A. S., Shemesh H., Tanomaru-Filho M., Wesselink P. R., Wu M.-K. (2009). The ability of different nickel-titanium rotary instruments to induce dentinal damage during canal preparation. *Journal of Endodontics*.

[24] De-Deus G., Cavalcante D. M., Belladonna F. G. (2019). Root dentinal microcracks: a post-extraction experimental phenomenon?. *International Endodontic Journal*.

[25] Arashiro F. N., De-Deus G., Belladonna F. G. (2020). Dentinal microcracks on freshly extracted teeth: the impact of the extraction technique. *International Endodontic Journal*.

